# Vulvar Epidermoid Cyst and Type 2 Radical Genital Mutilation

**DOI:** 10.1155/2015/520190

**Published:** 2015-11-22

**Authors:** Ozer Birge, Ertugrul Gazi Ozbey, Deniz Arslan, Mustafa Melih Erkan, Feyza Demir, Utku Akgor

**Affiliations:** ^1^Nyala Sudan Turkey Training and Research Hospital, Department of Gynecology and Obstetrics, Nyala, 7100 Darfur, Sudan; ^2^Nyala Sudan Turkey Training and Research Hospital, Department of Urology, Nyala, Darfur, Sudan; ^3^Celal Bayar University Hospital, Department of Gynecology and Obstetrics, Manisa, Turkey; ^4^Nyala Sudan Turkey Training and Research Hospital, Department of Pathology, Nyala, Darfur, Sudan

## Abstract

About 100 million women are estimated to be circumcised globally. Various rates of complications have been encountered, especially after circumcision, such as bleeding, infection, shock, menstrual irregularity, difficulty in urination or common urinary tract infections, inguinal pain, difficulty in sexual intercourse, and genital circumcision scar especially at the vulvar region, and cystic or solid character mass in short and long term. Furthermore, the maternal-fetal morbidity and mortality increase due to bleeding and fistula, which develop after prolonged labor, travail, and difficult labors. Our aim in this paper was to discuss a 42-year-old multiparous female case who had undergone type 2 radical genital mutilation (circumcision) when she was 7 years of age, along with the literature, which has been evaluated for the gradually growing mass at the left inguinal canal region in the last 10 years and diagnosed as epidermoid inclusion cyst developing secondary to postcircumcision surgical ground trauma, since there was no other case found in the literature search that had been circumcised at such an early age and developing after circumcision at such advanced age, and, therefore, this is suggested to be the first case on this subject.

## 1. Introduction

Mutilation of the female external genital organs causing disability or dysfunction (female genital mutilation or circumcision) includes interventions such as cutting, tearing off, stitching up, and cancellation by adhering to strict and wrong traditional rules for nonmedical reasons [1, 2]. Female genital mutilation is practiced in more than 30 countries, especially in Middle Africa, West Africa, and Southeast Asia [3]. Although it is utilized as the “so-called” female circumcision in the practiced countries, this practice is defined in medical practice as a word of Latin origin “mutylatio,” meaning mutilation and tearing off due to its unfavourable physical and physiological results [2, 4]. This practice is generally reported to be performed in infancy, childhood, puberty, and adolescent periods. Various rates of complications are encountered, especially after circumcision such as bleeding, infection, shock, menstrual irregularity, difficulty in urination or common urinary tract infections, inguinal pain, difficulty in sexual intercourse, and a genital circumcision scar, especially at the vulvar region, and a mass of cystic or solid character in the short and the long term [1]. While early period complications following female genital mutilation are more common, late period complications are seen in some rare cases.

Benign tumors of the vulva are rarely seen. Epidermoid cysts are slow growing benign cystic tumors surrounded by keratinized squamous epithelium and filled with keratin debris. Epidermal inclusion cyst, epidermoid cyst, and sebaceous cyst demonstrate the same pathology [5, 6]. Epidermoid tumors are intradermal or subcutaneous tumoral lesions of epidermis origin and, in particular, epidermoid cysts develop as a result of implantation of superficial epidermal tissue on dermal or subcutaneous tissue following trauma or surgical procedures [6, 7]. Epidermoid cysts can be localized at any part of the human body, especially inside the mouth, in the extremities, and on the scalp when exposed to trauma; however, they are rarely seen in the vulvar region [8, 9]. Epidermoid vulvar cysts are generally multicystic and slow growing and their growth rates slow down after reaching a size of 5 × 6 cm and continue to grow in chronic grounds. Histopathologically, they differ from other vulvar lesions. Furthermore, even in large cystic lesions, very good cosmetic and cure results are obtained following total mass excision [10, 11]. It has been defined in the literature that large epidermoid cysts involving the vulva, vagina, and clitoral regions can develop after many years secondary to a surgical traumatic event following female genital mutilation [12].

In this case, there was a type 2 radical circumcision history at an early age and then a mass located in the inguinal region, growing with age, was detected. The case, which has been reported as epidermoid inclusion cyst in the histopathological examination, has been discussed in this presentation.

## 2. Case Presentation

**Figure 1:**
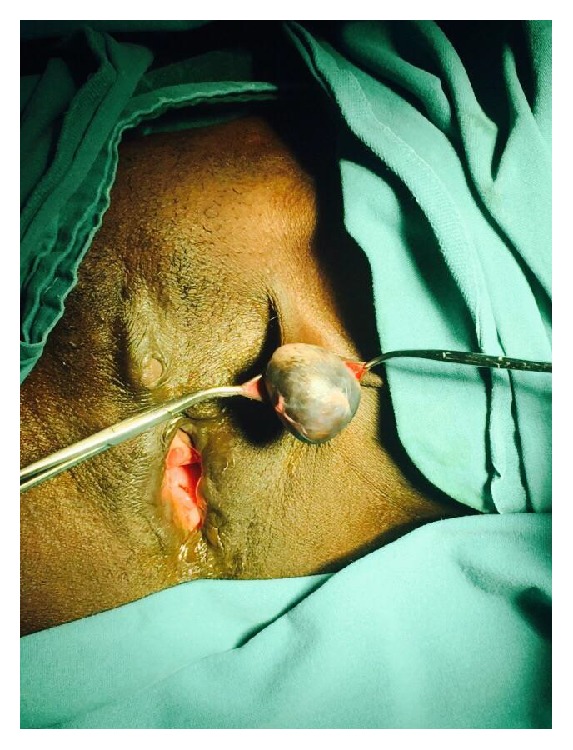
Preoperative inspection of left vulvar region.

**Figure 2:**
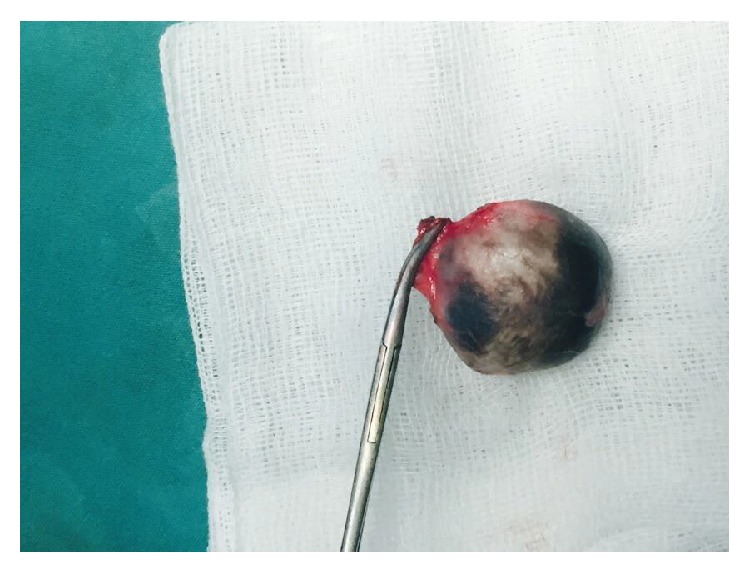
Postoperative image of the cyst after removal (filled with off-white granulose keratinous build-up).

**Figure 3:**
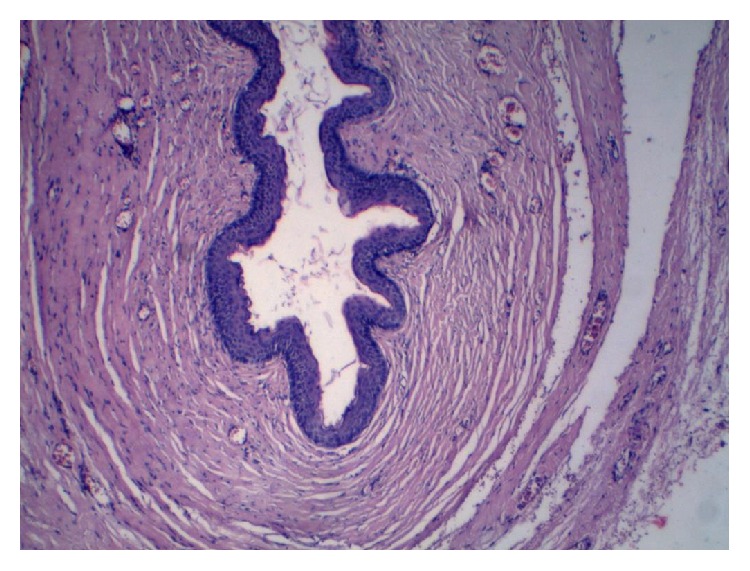
Histopathological appearance of the epidermoid cyst lined by stratified squamous epithelium and filled with keratinous material (H&E ×40).

A 42-year-old multiparous female case was admitted due to a palpable itching mass in the genital region that caused difficulty in walking and difficulty in sexual intercourse. It had been reported in the history of the case that she had been circumcised by a village midwife at the age of 7, who was brought by her mother, and then she got married at the age of 16. She delivered through vaginal delivery 3 times at home; the small papillary formation that is present at her left inguinal region that has caused itching from time to time since her puberty has grown rapidly in recent years, eliciting pain in the genital region and difficulty in sexual intercourse. She presented to the obstetrics and gynaecology clinic compulsorily at the request of her husband and, furthermore, she has not had a child for the last 5 years due to the problem in sexual intercourse and she stated that this situation affected her family life. On the evaluation of the case, a 4 × 3 × 2 cm sized, regularly contoured, mobile, sensitive mass was visualized, which was covered with normal skin, including a cystic structure, at the left vulvar inguinal region that caused difficulty in walking and sitting. In the detailed assessment of the case, it was observed that the mass had a cystic component and there was a previous type 2 genital circumcision scar close to the border of the labium majus on the skin of the left inguinal canal (Figure 1). The routine complete blood count, complete urine analysis, and biochemistry parameters were within normal limits, and the case was evaluated as a benign appearing mass of cystic nature without lobulation and septation on the performed superficial tissue ultrasonography. The case underwent an elective total mass excision operation. Perioperatively, a 4 × 3 cm sized cystic mass, filled with keratinous material in the slices, of off-white colour, was observed (Figure 2). On microscopy, squamous epithelium consisting of a cyst wall and keratinous material inside the cyst lumen (H&E 40) were detected (Figure 3). Epidermoid cyst was reported on the histopathological examination of the patient, who had no other trauma and surgery history other than genital circumcision in the vulvar region. The patient had no problems in the postoperative period and the 3rd month control was stated to be normal, and it was also stated that she did not have any anatomical and functional problems and that they had no problem during sexual intercourse and, therefore, she and her spouse were feeling happy.

## 3. Discussion

The mutilation procedure is currently practiced in thirty African countries, in several countries in the Arabian peninsula, in some communities in Southeast Asia, and secretly in ethnic communities that have migrated from these countries to Europe, America, and Australia [2, 13]. It has been observed that female genital mutilation operations have also been practiced in Western countries from time to time throughout the history [13]. Although the historical origin of this traditional practice is not precisely known, it is believed to exist since the ancient Egyptian civilization. According to the reports of the World Health Organization, it has been reported that approximately 100–150 million living women have been exposed to this practice and that this practice is applied on 6000 African girls between the ages of 4–12 years every day, and two million new applications are performed annually in the world [2, 14]. Circumcisions are generally performed under primitive and traditional conditions and applied differently in various countries; despite the fact that this procedure, which aims at realization of sexuality in later life and affects the psychological and physiological sexual lives in women in an extremely negative way, was banned in many countries with the efforts of the World Health Organization, it still continues to be secretly practiced in rural areas.

The World Health Organization has divided circumcision types into four different groups for classification [2, 4, 15]:

 Type 1: partial or total removal of the clitoris with preputium (Sunna).
 Type 2: removal of preputium and labium minor and partial removal of labium major (excision).
 Type 3: almost total removal and cutting of clitoris and preputium together with labium minor and labium major and suturing the open wound sites by bringing them together and leaving a small opening for flow of menstrual bleeding and urine (infibulation).
 Type 4: a new group that includes all interventions aiming at mutilation (pricking, piercing, incising, scraping, and cauterizing); however, also in the previous classification, this type is the most severe one and includes all the previous types and results from partial removal of the vagina, and it was called the Pharaoh's circumcision, since it was applied during the ancient Egyptian pharaoh era.

Type 1 and type 2 female genital mutilations comprise 80% of all genital mutilation cases [15]. However, the current case we are reporting from the Darfur region of the Middle Africa country of Sudan is a type 2 genital mutilation.

The female genital mutilation procedure is performed without applying any anesthesia or hemostasis process under nonsterile conditions, particularly by older women popularly described as midwives [16]. Bleeding, hematoma, inability to urinate, inability of sexual intercourse, severe pain due to genital tissue damage, tetanus, sepsis, and death due to shock are seen, especially following the acute period of the mutilation. The long term complications are frequent urinary tract infections, infertility, sexual problems, increase in postpartum complications, epidermal cyst-like lesions, and an increase in psychological problems as a result of the aforementioned [17, 18]. Epidermoid cysts are benign multilobulated cystic lesions that can be seen at any area of the human body including the epithelium layer, especially in areas exposed to trauma such as the face, neck, inner surface of the ear, hand and foot, back, extremity, and scalp [5, 6, 8, 9]. In closed ethnic societies, in particular, it is seen as a mass in the chronic period as a complication of a surgical or traumatic procedure following female genital mutilation. Following female genital mutilation, it is particularly seen in the clitoris, labium majus, and the labium minus at various rates according to the type of the circumcision [10, 11]. Since vulvar epidermoid cysts developing in the long run following female genital mutilation are generally painless, either the cases can be mostly asymptomatic or the patient may present with nonspecific complaints such as the inability to urinate, vaginal discharge, palpable mass in vulva, and chronic pain in vulvar region [16, 19]. While epidermoid cysts in the genital region generally develop as a result of a traumatic event or following a surgical procedure, rare cases that develop without trauma or surgical procedure have also been reported [19, 20]. On review of the literature, the largest epidermoid mass that has developed in the labium major following trauma was reported by Yang et al. and was about 12 cm in size and had approximately 10 years of clinical history [21]. The issue of the prevalence of epidermoid cyst development and when it can develop following female genital mutilation varies and unmet estrogenic effects due to anovulatory cycles are thought to be effective on this development [22]. In the presented case, there was no similar case encountered in the literature in advanced age as a case of a regularly menstruating 42-year-old woman. In a case reported from America in 2010, the case of a type 1 female genital mutilation performed on a 37-year-old Afro-American woman who had difficulty in walking was reported, in which the patient had undergone an examination due to a palpable mass of a postmutilation epidermoid cyst that had been present for the last 6 months, which was of clitoral origin, which was discovered on examination after 30 years [23].

In our epidermoid clitoral cyst case, a radical type female genital mutilation, which is a surgical procedure, had been performed on the genital region as a tradition of her own ethnic community in early childhood. Difficulties in walking and sexual problems were found in our case, despite the fact that she wanted to have a child for a long time, and this was suggested to be due to the sensitivity of the mass and the aesthetically poor appearance. The main treatment for epidermoid inclusion cyst is total removal of the cyst and postoperative recurrence is not reported. Total cyst removal was performed in the presented case and there has been no recurrence symptom on the follow-up.

## 4. Conclusion

Consequently, female genital mutilation is still widely applied today in Africa and Asia, especially in culturally and traditionally closed societies. Similar publications are required for detailed treatments of short and long term complications due to the surgical traumatic effect following female genital mutilation, particularly in those performed at early ages.
